# Elastic Wave Monitoring of Cementitious Mixtures Including Internal Curing Mechanisms

**DOI:** 10.3390/s21072463

**Published:** 2021-04-02

**Authors:** Gerlinde Lefever, Didier Snoeck, Nele De Belie, Danny Van Hemelrijck, Dimitrios G. Aggelis

**Affiliations:** 1 Department of Mechanics of Materials and Constructions, Vrije Universiteit Brussel (VUB), Pleinlaan 2, 1050 Brussels, Belgium; Didier.Snoeck@vub.be (D.S.); Danny.Van.Hemelrijck@vub.be (D.V.H.); Dimitrios.Aggelis@vub.be (D.G.A.); 2 Magnel-Vandepitte Laboratory for Structural Engineering and Building Materials, Department of Structural Engineering and Building Materials, Faculty of Engineering and Architecture, Ghent University, Tech Lane Ghent Science Park, Technologiepark Zwijnaarde 60, 9052 Ghent, Belgium; Nele.DeBelie@UGent.be

**Keywords:** acoustic emission, ultrasound, cement, internal curing, superabsorbent polymers, hydrogels, lightweight aggregates

## Abstract

The mitigation of autogenous shrinkage in cementitious materials by internal curing has been widely studied. By the inclusion of water reservoirs, in form of saturated lightweight aggregates or superabsorbent polymers, additional water is provided to the hydrating matrix. The onset of water release is of high importance and determines the efficiency of the internal curing mechanism. However, the monitoring of it poses problems as it is a process that takes place in the microstructure. Using acoustic emission (AE) sensors, the internal curing process is monitored, revealing its initiation and intensity, as well as the duration. In addition, AE is able to capture the water evaporation from saturated specimens. By ultrasonic testing, differences in the hydration kinetics are observed imposed by the different methods of internal curing. The results presented in this paper show the sensitivity of combined AE and ultrasound experiments to various fundamental mechanisms taking place inside cementitious materials and demonstrate the ability of acoustic emission to evaluate internal curing in a non-destructive and easily implementable way.

## 1. Introduction

High-performance cementitious materials present an increased mechanical strength and a higher durability compared to conventional concrete. The combination of these aspects allows for larger structures to be built and promotes sustainability in construction industry. However, these mixtures are characterized by a low water-to-cement (w/c) ratio, which leads to self-desiccation of the matrix and autogenous shrinkage. When shrinkage is restrained, internal stresses develop within the material and induce shrinkage cracking. As these cracks might harm the future functioning of the structural element, autogenous shrinkage needs to be mitigated.

To counteract self-desiccation, water should be provided to the mixture. A method that is studied in-depth is internal curing [1,2,3,4]. In this technique, extra water is added during mixing, but not in direct contact with the cement particles, as this would explicitly increase the w/c ratio. Therefore, additives are used that act as internal water reservoirs. During hydration, the free water is consumed, which causes a drop in the relative humidity and an increase in the capillary pressure [5,6]. Whereas this elevated capillary tension is the main cause of the autogenous shrinkage development in cementitious mixtures, it is also the driving force for the activation of the internal curing mechanism. The capillary suction removes the water from the included reservoirs, allowing for continued hydration to take place. In this way, the relative humidity can be maintained, and autogenous shrinkage is counteracted. In literature, mainly two additives are investigated: saturated lightweight aggregates (LWAs) and superabsorbent polymers (SAPs).

In case of saturated LWAs, the additional water for internal curing purpose is absorbed by the porous aggregates before their inclusion into the cementitious blend. In a study of Cusson and Hoogeveen [7], saturated lightweight sand was investigated. By replacing part of the fine aggregate by pre-saturated porous aggregates, the extent of free shrinkage strain was reduced. Additionally, the decrease of shrinkage showed to be proportional to the amount of internal curing provided. Bentur et al. [2] replaced part of the normal-weight aggregates by a commercial LWA. They concluded on the effective mitigation of autogenous shrinkage, but at the cost of a lowered compressive strength. Various studies reveal the beneficial effect of LWAs for internal curing [3,8,9,10,11]. However, care should be taken on the type of aggregate to be used for a certain mixture, as the ideal amount of water to be added for internal curing depends on the absorption capacity of the aggregate and on the water content of the cementitious mix [4]. Similar to the addition of LWAs is the use of SAPs. As their name implies, these polymers are able to absorb and retain large quantities of water. Whereas the absorption capacity of LWAs amounts to few percent of their initial dry weight, SAPs can absorb up to 500 times their own weight. Moreover, in case of LWAs the saturated aggregate is added during mixing, SAPs are mostly included in the dry state and absorb water during the mixing process. The amount of water that is absorbed by the SAPs during mixing is then added on top of the mixing water [12]. Several researches have already studied the effect of SAPs on the development of autogenous shrinkage and confirmed their positive effect on mitigating both shrinkage and cracking [13,14,15,16].

Both LWAs and SAPs offer a solution for mitigating autogenous shrinkage in cementitious materials. However, the question arises whether any type of porous aggregate or SAP provides effective internal curing and how these additions exactly influence the hardening process. As already mentioned, the storage of water within these reservoirs is necessary to maintain the water-to-cement ratio of the mixture. When the water is released too early, i.e., before final setting, the w/c ratio may increase, leading to a lowered mechanical performance. On the contrary, when the water is released too slow, autogenous shrinkage might have initiated and the risk of shrinkage cracking is increased. The moment of water release by the LWA or SAP is thus of high importance. Studies concerning the kinetics of water sorption and desorption of SAPs describe the use and relevance of neutron radiography [17] and nuclear magnetic resonance (NMR) [18,19]. However, these tests are not widely accessible and available due to their high cost and cannot be combined with the measurement of autogenous shrinkage. In a recent study, the technique of acoustic emission (AE) monitoring was utilized to expose the water release by the SAPs [16]. Two piezoelectric sensors, attached to an instrumented steel ring for autogenous shrinkage measurements, captured an elevated amount of hits closely after setting, revealing the onset of internal curing. A deeper investigation of the received wave parameters showed that the internal curing mechanism exhibited a peak intensity period, likely during which most of the water is released to the surrounding matrix [20]. AE therefore provides a non-destructive, easily applicable technique to monitor the internal curing process.

Secondly, whereas the working principle of LWAs and SAPs is similar, some differences between both additives are noticed. Firstly, the saturated LWA absorbed water before its addition to the cementitious mixture, while SAPs absorb water during mixing and thereby dilute the ion concentration of the fresh material. Additionally, the inclusion of lightweight aggregates creates stiff, but porous localized zones inside the hardened matrix, whereas the SAPs shrink after releasing their absorbed water and simply leave behind pores. Most likely, these additives will therefore affect the hydration process in their own way, leading to a variation in setting times and changes in the developed microstructure and the macrostructural properties.

In this study, AE was used to monitor the hydration process of cementitious mixtures with and without internal curing mechanism. Over the last decades, this technique has been extensively used and showed to be sensitive to various processes occurring inside cementitious materials, such as particle settlement [21], hydration reaction [22,23] and fracture [24,25]. By analyzing the AE data, the time period of water release by lightweight aggregates and superabsorbent polymers is defined and different occurring processes, such as settlement, hydration and internal curing, are separated. AE results allow to evaluate the efficiency and enable comparisons between the included curing mechanisms (LWA and SAPS) to mitigate autogenous shrinkage for the first time. In an effort to determine the actual source of AE, monitoring experiments were also conducted on hardened specimens after a saturation period, using sensors attached directly to the specimen’s surface. These measurements indicated that AE is also sensitive to the evaporation of water from the porosity of cement-based media. In addition, within this testing series, cement paste and mortar mixtures were investigated in order to distinguish between different cementitious media with increasing level of complexity. Next to the AE experiments, ultrasound (US) measurements were conducted on mortar blends to supply additional information on the effect of LWA and SAPs on the stiffness development in real time.

## 2. Materials and Methods

### 2.1. Mixture Design

Three cement pastes and their equivalent mortar blends were studied: a reference mixture, a mixture with lightweight aggregates and a mixture with SAPs. The type of cement used is CEM I 52.5 Strong (Holcim, Nivelles, Belgium), which is a high-strength Portland cement, containing at least 95% of Portland clinker. The water-to-cement ratio of all mixtures is equal to 0.35. In case of mortar blends, oven-dried river sand 0/2 is added, using a sand-to-cement ratio of 2. To increase the flowability and facilitate casting of the specimens, superplasticizer MasterGlenium 51 (BASF, Ludwigshafen, Germany) is included in an amount of 0.4% by weight of the cement.

The SAP used is a copolymer of acrylamide and sodium acrylate, provided by BASF (Ludwigshafen, Germany). This superabsorbent polymer has a particle size of 100 ± 21.5 µm [14] and presents the ideal characteristics for internal curing purpose. An amount of 0.2% of SAP with respect to the cement quantity was included together with an additional amount of water equal to 26 g per gram of SAP, which is the absorption capacity in cementitious environment, based on previous research studies [16,26]. Adding these quantities of SAP and additional water, an identical workability of the SAP mortar compared to the reference material was obtained, meaning that the additional water volume is completely taken by the SAPs.

As a lightweight aggregate, Granulège (Cellumat, Saint-Saulve, France) was investigated. These aggregates are crushed aerated concrete particles, consisting of calcium silicate hydrates and sand, and are often used for thermal insulation layers. The particles have a density of 290 kg/m³ and a diameter in between 1 and 8 mm. Their porosity allows for a water absorption of 100 ± 15%, which was obtained after submersion in water for 24 h. To be able to compare the results between SAP and LWA mixtures, an identical amount of entrained water was included into both blends. Having an absorption capacity of 1 g of water per gram of LWA, the ratio of LWA to cement was equal to 5.2%.

A summary of the mixture compositions can be found in Table 1 for mortar mixtures. From this point onwards, the use of the nominations reference, SAP and LWA in tables and figures denotes the cement paste or mortar mixture without additives, with SAPs and with LWAs, respectively. The amount of LWA presents the weight of the dry LWA. The water necessary to saturate the aggregates is added to the water content. In case of cement pastes, the sand was omitted from the mixtures.

The density and the compressive strength of the three mortar mixtures after 28 days of curing are summarized in Table 2. In order to obtain these properties, three prism specimens per mixture, measuring 40 mm × 40 mm × 160 mm, were cast and cured in plastic foil at a temperature of 21 ± 1 °C. The density was obtained by weighing the cured specimens and measuring their exact dimensions, while the compressive strength was obtained following ASTM C349-18 [27], performed on the remaining prism halves obtained after a three-point bending test.

Regarding the volumetric mass of mortar mixtures, it can be seen that the inclusion of saturated LWAs significantly lowered the density of the cementitious mixture. Whereas an identical volume of absorbed water was added in SAP and LWA blends, the empty porosity of LWA mixtures must be much higher compared to the SAP mortar after 28 days of curing. This could indicate that the used lightweight aggregates contain pores that are not accessible for the water or that a relatively large fraction of the aggregates’ porosity is not able to retain the water, but releases the water immediately after removal of the LWAs from the water bath. The reduction in density was also confirmed by the strong decrease in compressive strength, being about 20%, from the reference mortars to the LWA mixtures. In case of the SAP inclusion, a more limited reduction in compressive strength compared to the reference was observed. Similar to the mixtures with LWA, this decrease in mechanical performance is caused by the porosity, and in this case the creation of macropores as the water is released from the SAPs. Nonetheless, the density of SAP mortars was approximately identical to the reference material. This increased density of the SAP mixtures compared to the LWA mortars could also be caused by the probably more porous interfacial zone around the lightweight aggregates. Moreover, due to their smaller size, the SAP particles are more homogeneously distributed compared to the LWAs. When the water is released, internal curing is provided to the zone around the water reservoirs. The area reached is therefore higher for reservoirs with a larger surface area, so that the SAPs induce more effective curing compared to the saturated aggregates, increasing the density of the mortar material in the cured zones.

### 2.2. Test Methods

#### 2.2.1. Acoustic Emission

**Figure 1:**
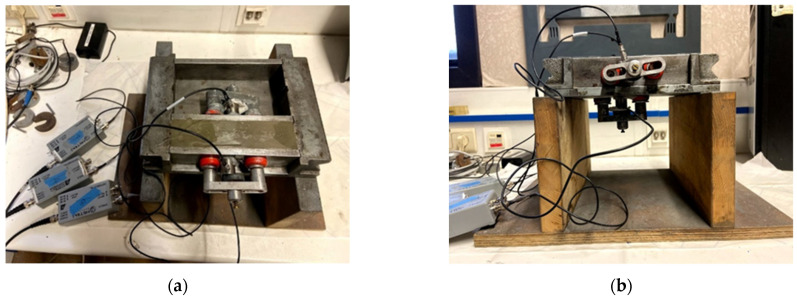
Acoustic emission set-up for fresh monitoring: (**a**) top view showing two opposing sensors attached to the longitudinal walls by means of magnetic holders and (**b**) side view showing the bottom sensor (reprinted from [20]).

The hydration and internal curing processes were monitored using three piezoelectric R15α sensors, mounted on a metallic, prism-shaped mold measuring 40 mm × 40 mm × 160 mm. Two of these sensors were attached to the longitudinal walls, whereas the third one was placed on the bottom surface (Figure 1). The operating frequency band of the sensors is between 50 and 400 kHz and the resonance frequency is 150 kHz. After casting, the samples were covered by means of a thin plastic foil to avoid evaporation and the monitoring was continued for up to four days.

In order to obtain more information on the actual sources of AE during internal curing, the monitoring was repeated on hardened, saturated specimens in open air. As these samples are drying, water evaporation takes place in all mixtures, also the reference material. In this way, the water mobility could be tracked by AE and the activity could be compared in between mixtures with and without internal curing mechanism. When the first (fresh) monitoring stage was finished at an age of 4 days, one sample per mixture composition was demolded and left to open air conditions until the age of 14 days and afterwards placed in water for 48 h. The samples’ weight increase was then measured after pat drying the surfaces with a damp cloth. Consequently, three R15α sensors were attached to the specimens’ surface with Vaseline and paper tape, in similar locations compared to the fresh monitoring experiment. AE monitoring was conducted for 48 h in an air-conditioned room at a temperature of 21 ± 1 °C.

#### 2.2.2. Ultrasound

**Figure 2:**
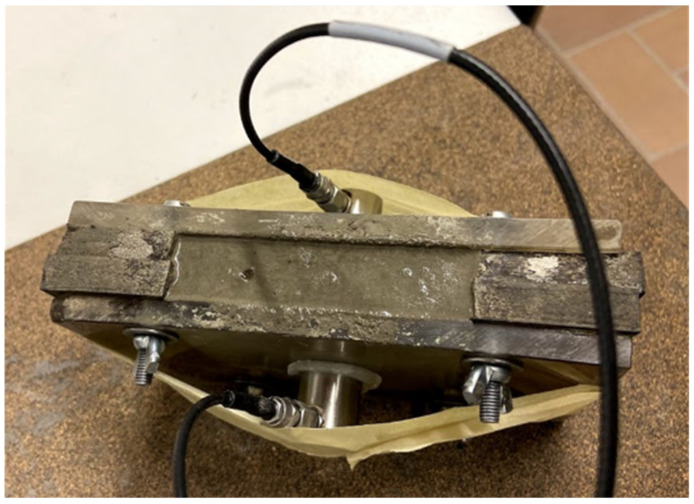
Ultrasound set-up showing the mortar sample in between both sensors.

The follow-up of the hardening process and the determination of the initial and final setting time were conducted by means of ultrasound measurement on fresh mortar specimens. The method adopted within this research is based on the RILEM recommendation TC 218-SFC [28,29] and is based on the transmission of elastic waves. The mold, shown in Figure 2, consists of two plexiglass walls with a rubber sheet in between. Within the latter, a U-shaped opening is made, in which the mortar can be cast. A longitudinal sine wave with a frequency of 150 kHz and an amplitude of 10 V was sent through the plexiglass walls and the mortar specimen by means of a piezoelectric R15α sensor. On the other side of the mold, this signal is received by a second, identical sensor. As the fresh mortar hardens, the wave velocity, measured based on the first threshold crossing, increases over curing time. By an analysis of the velocity vs. time curves, variations in the hydration kinetics between different mixtures can be revealed and the initial and final setting times can be determined [30]. The detailed procedure used to determine the initial and final setting time can be found in [31].

## 3. Results and Discussion

### 3.1. Acoustic Emission Monitoring of Cement Pastes

**Figure 3:**
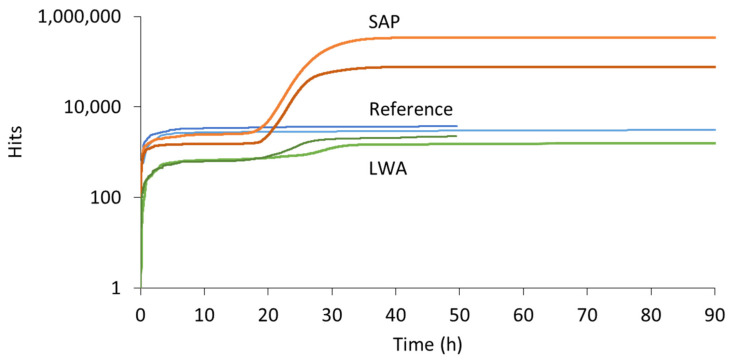
Cumulative hits vs. curing time received during AE monitoring of reference (= blue), SAP (= orange) and LWA (= green) cement paste.

The cumulative hits received during the AE monitoring of cement paste specimens are shown in Figure 3. For every mixture, two replicates were made. The colors distinguish the different mixtures (reference = blue, SAP = orange, LWA = green), whereas the shading is used to separate the replicates. This color code is maintained during the entire discussion to facilitate the comparisons made. Clearly, the mixtures with SAPs show a much higher activity compared to both reference and LWA mixtures. This increased amount of hits received was not seen from the start of the experiment, but initiated in between 10 and 20 h of curing.

During the first two to four hours, an increased activity was seen for all mixtures, which can be linked to particle settlement [21]. Afterwards, the activity slowed down and for several hours of curing the hit rate remained fairly constant. In case of the reference mixture, this period of low activity continued throughout the entire experiment, i.e., until three days of curing. For the SAP pastes, the hit rate changes after 16 to 18 h of curing, depending on the tested replicate. A correlation with the time of water release by the SAPs, inducing internal curing, was found in literature [19]. In the latter study, it was seen that the SAPs under study retained their absorbed water until final setting of the cementitious mixture, after which the water was gradually released. The AE activity received during this time period can thus be linked to the activation of the SAPs. Similarly, the mixtures with saturated LWAs show an increased activity after about 18 to 24 h of curing, signifying internal curing. The onset of internal curing was thus slightly delayed compared to the SAP specimens. It was seen that, in contrast to all other cement paste and mortar mixtures (also mortar including LWA), bleeding occurred to a small extent. It is assumed that the LWAs released part of their absorbed water quickly, due to their open porosity. Thereby the w/c ratio was effectively increased, meaning that the relative humidity of the cement paste could be maintained for a longer time. Consequently, the capillary pressure increases at a reduced rate compared to the SAP blend, which leads to a later withdrawal of the absorbed water.

A further comparison between SAP and LWA mortars displays a change in the number of hits received and the duration of the internal curing action as well. In LWA mixtures, the total amount of hits related to internal curing was up to 100 times smaller compared to SAP blends. Additionally for LWAs, the hit acquisition decelerated quickly, i.e., after six hours of increased activity, whereas for SAP blends the highest hit rate was maintained for 10 h, leading to a total SAP activity period of about 20 h. This result illustrates some interesting differences in between both additives. Firstly, LWAs release their absorbed water at a high rate, which is less ideal for internal curing purposes. This is caused by the open porosity of the LWAs, compared to the hydrophilic polymer chains, which allow for an easier retraction of the absorbed water, of the SAPs. Secondly, after the water is removed from the LWAs, the material becomes “quiet” again, while for SAP mixtures the activity is due to an alternation of water release and SAP shrinkage, leading to friction between the SAP particle and the pore wall. The latter is most likely the cause of the increased number of captured hits in SAP mixtures. Lastly, it should be noted that, while the activity of SAP mixtures was increased in comparison to reference and LWA pastes, the number of hits received is not identical for the two replicates. However, a similar trend could be distinguished when comparing both cumulative hit curves.

**Figure 4:**
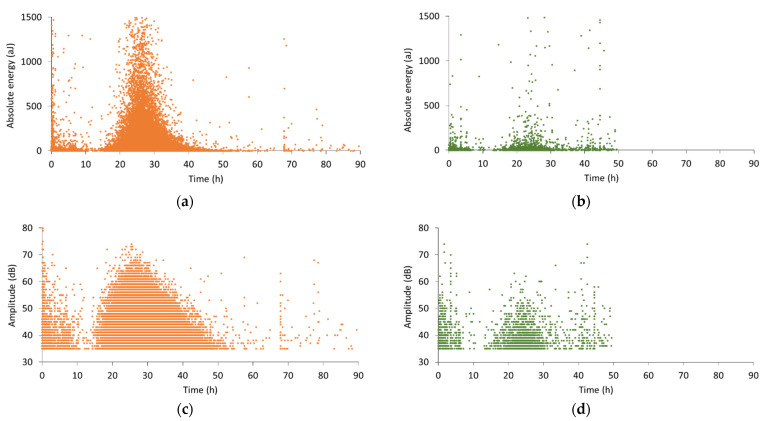
Wave parameter analysis: absolute energy of (**a**) SAP and (**b**) LWA paste compared to amplitude of (**c**) SAP and (**d**) LWA paste.

To obtain more information regarding the internal curing process in SAP and LWA mixtures, an analysis of the wave parameters, such as the absolute energy and the amplitude, was performed. In Figure 4, the absolute energy and amplitude of all hits received during monitoring of a representative SAP and LWA sample are plotted. The used colors represent the specific replicates, identical to Figure 3. Regarding the absolute energy of SAP-related signals (Figure 4a), it is seen that a large variety of energy values was captured during internal curing. However, the phenomenon unfolds mainly between 16 and 25 h, with a peak at approximately 25 h, where peak energy values reach close to 1500 aJ. This demonstrates that internal curing is not a constant process, on the contrary, its intensity varies with respect to the curing time. Similarly, the amplitude in Figure 4c increases rapidly within the same time period. At around 25 h of curing, amplitudes of 75 dB are observed. Afterwards, the peak amplitude decreases again along with a reduction in the hit rate. Whereas the cumulative hits in Figure 3 depict a slow continuation of the activity after 40 h of curing, the amplitude vs. time graph suggests the end of internal curing after approximately 50 h of curing. Besides this large cluster of received hits, a considerable amount of hits is captured before and after internal curing. During the first hours of curing, high absolute energy as well as high amplitude are noticed, linked to particle settlement, while afterwards both signal parameters are lowered before they increase again due to internal curing.

For the signals received during internal curing by LWAs, the absolute energy showed to vary as well, but with a much lower number of acoustic hits (Figure 4b). Compared to the SAP mixtures, the energy levels remained rather low, i.e., mostly below 200 aJ. Concerning the signal amplitude in Figure 4d, an increasing trend was observed from the onset of water release until 24 h of curing. Later on, the amplitude values reduced quickly together with the decelerated activity. When comparing these results to the amplitude of SAP-related signals, it was noticed that the latter values were much higher, reaching up to 75 dB, whereas in LWA pastes only a limited number of received waves showed an amplitude above 60 dB.

Next on, as aforementioned, and after a curing age of 14 days, one sample of each composition (reference, SAP and LWA) was placed in water for 48 h. Their weight was measured before and after saturation, and is denoted in Table 3. It can be observed that the water uptake of reference and SAP samples lied closely to one another, while the LWA specimen absorbed a much larger quantity of water. This increased absorption capacity is most likely due to the higher porosity of LWA cement pastes (and mortars), which could be seen from the reduced density in Table 2. While one would expect to have an increased water absorption for the SAP specimen compared to the reference too, the similar water uptake suggests that the SAP-related porosity is less accessible from the outer environment, i.e., the pores are discrete rather than interconnected.

**Figure 5:**
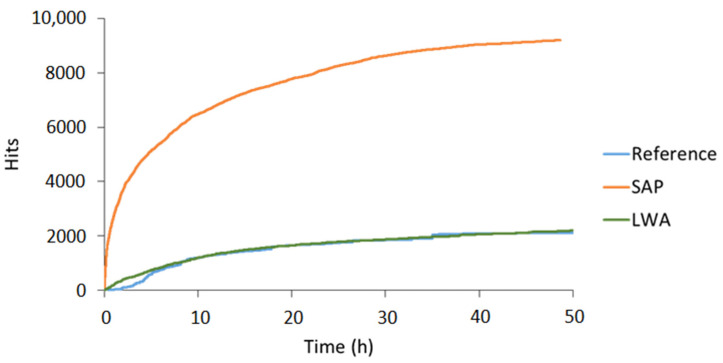
Cumulative hits vs. evaporation time of saturated reference, SAP and LWA cement pastes.

AE sensors were again applied on the specimens during the water evaporation stage. In this case, three piezoelectric sensors were attached directly to the specimen surfaces. It was found for the first time in literature that AE is also sensitive to the evaporation process. The cumulative hits from different specimens are depicted in Figure 5. All curves show a continuously decreasing slope, but obviously the curve of reference and LWA is much lower positioned than SAPs. Whereas a discrepancy was seen between the water uptake of reference and LWA samples, the amount of hits received from both specimens was almost identical and remains rather low. On the contrary, in case of SAP inclusion, the number of captured hits was higher immediately from the start of the experiment, although the water absorption was similar to the reference. This implies that a different source, apart from water mobility and evaporation, takes place. This source can be attributed to the shrinkage of the SAPs and detachment or friction against the pore walls and seems to constitute the main source of AE during water release by SAPs.

### 3.2. Acoustic Emission Monitoring of Cementitious Mortars

**Figure 6:**
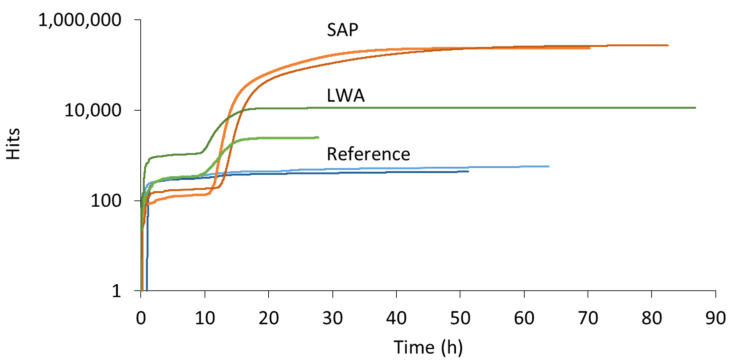
Cumulative hits vs. curing time received during AE monitoring of reference (= blue), SAP (=orange) and LWA (=green) mortar.

To check one higher level of material complexity, mortar mixtures were also used. Two reference, SAP and LWA mortar replicates were cast and monitored by means of AE. In Figure 6, the cumulative hits are plotted with respect to the curing time. Similar to the cement pastes, SAP mortars exhibit a significantly higher activity compared to reference and LWA specimens. Again, this activity was mainly captured after several hours of curing, demonstrating the onset of internal curing.

In comparison to the cement pastes, where up to 3500 hits were received during the settlement period, only one of the mortar samples revealed a noteworthy amount of hits during the first two hours. Due to the addition of sand in the latter mixtures, the liquidity of the freshly mixed material is drastically decreased, reducing the ease of particle settlement. Focusing on the reference replicates, a fairly constant hit level was maintained throughout the entire experiment, comparable to the result of the reference cement pastes. For the mortars with internal curing mechanism, an increase in AE activity was found after approximately half a day of curing. In case of the SAP mixtures, the initiation of the water release was observed after 11 to 12 h of curing. In comparison to their equivalent cement pastes, this onset was advanced by several hours, which is caused by the reduced humidity of mortars compared to pastes. As sand is added, these (oven-dried) particles attract and adsorb water, limiting the volume of free water present for hydration purpose. The relative humidity of the mixture therefore decreases earlier and, consequently, the capillary pressure rises sooner and extracts the water from the SAPs. An increased hit rate was noticed until two to three days of curing, depending on the replicate. Likewise, an increased amount of hits was captured in LWA mortars and this after approximately 10 h of curing. Whereas this activity was also earlier compared to the onset of internal curing in LWA pastes, its starting point is now much closer to the one of SAP mortars, even a little earlier. This phenomenon supports the early, partial release of water by LWA in cement paste blends, which led to an increased w/c ratio and a delay in internal curing. Within mortar mixtures, the saturated LWAs held the water more effectively, as no bleeding was noticed. This was demonstrated by the workability test performed upon the design of the mixture compositions, showing an identical flow for reference, SAP and LWA mortars, and the fact that no bleeding took place.

**Figure 7:**
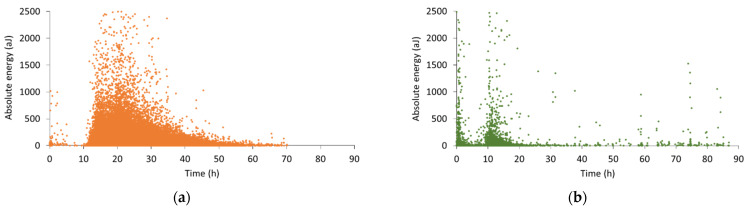

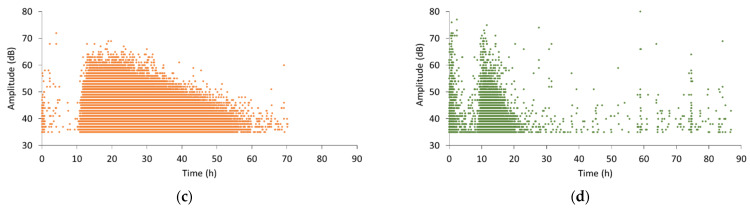
Wave parameter analysis: absolute energy of (**a**) SAP and (**b**) LWA mortar compared to amplitude of (**c**) SAP and (**d**) LWA mortar.

In Figure 7a–d, the absolute energy and amplitude of the received wave signals of a SAP and LWA mortar are shown. Similar to the SAP cement pastes, the waves’ absolute energy fluctuates during the internal curing process in mortar specimens. However, the absolute energy increases rapidly within the first two hours of internal curing (11 to 12 h), in contrast to the monitored cement pastes, where a more gradual increase over time was observed. This level of high absolute energy is maintained for about 10 to 15 h and decreases afterwards. Moreover, higher energy levels were reached compared to the cement paste, which can be related to the increased stiffness as sand is added. The amplitude, depicted in Figure 7c, displays the same trend: few hours after the onset of internal curing, the peak amplitude values reach up to 65 dB and until 25 h of curing. Later on, the maximum amplitude exhibits a gradual decrease to the level of 40 dB.

An analogous evolution of the absolute energy and amplitude can be noticed for LWA-related signals. Both parameters show an almost immediate increase at the start of internal curing, which was not seen for LWA pastes. Besides the dissimilarities between mortars and cement paste, some correspondence can be seen between these two cementitious materials. Firstly, the analysis of the wave parameters demonstrates that the internal curing process is not constant, this in both mortar and cement paste. Secondly, the difference between SAP and LWA inclusion for internal curing was confirmed. The monitoring of mortars as well as cement pastes displays the prolonged activity for SAP mixtures compared to LWA blends, which is caused by the more gradual release of water by the SAPs and the shrinkage of the SAPs inside the pores. For this reason, the use of AE was concluded to be valuable for the non-destructive monitoring of the internal curing action, providing information on the period of water release by the saturated reservoirs. In this way, the AE technique can be used to investigate whether a specific type of SAP or LWA is efficient for internal curing purpose, without the need of expensive measuring techniques such as neutron radiography and/or NMR.

### 3.3. Ultrasound

**Figure 8:**
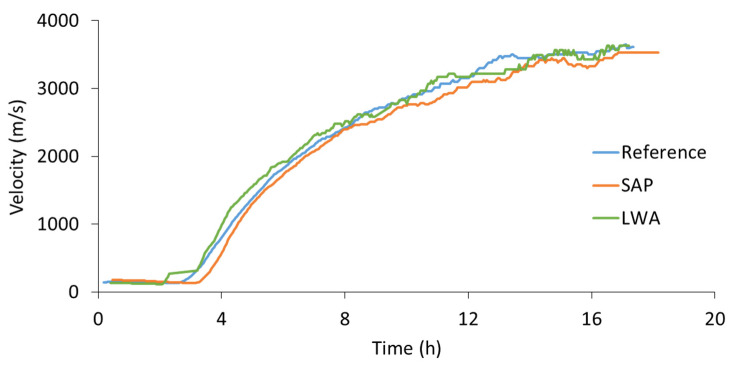
Velocity vs. time of reference, SAP and LWA mortar.

While AE was able to reveal the settlement (first two to three hours) and the internal curing process, no information was provided during the “silent” period in between these two stages. On the other hand, ultrasound experiments show no variations during the first hours of curing, but reveal the developing stiffness later on. Therefore, indicative ultrasound monitoring was conducted on fresh mortar mixtures, as described in Section 2.2.2, to complement the AE monitoring results and obtain a complete examination of the processes occurring inside fresh cementitious materials. In Figure 8, the velocity of the transmitted waves is plotted with respect to the curing time for one of the two replicates tested per mixture. Time zero represents the moment of first contact between water and cement. During the first hours of curing, the velocity of all mixtures remains at a constant level of approximately 150 m/s. Within this time frame, closely linked to the dormant period, the mortar remains deformable and settlement was captured by AE monitoring. After approximately three hours of curing, an increase in the wave velocity is noticed for the reference blend. The rising velocity is caused by the development of a stiff skeleton, as the main hydration reaction takes place. It can thus be observed that ultrasound discloses the hydration process in a more detailed way compared to AE, where only a small number of hits was captured.

Regarding the SAP mortar, a short delay can be seen compared to the reference material, which is caused by a diluted ion concentration. As the SAPs absorb water during mixing, the initial ion concentration is reduced, which slows down the hydration reaction and also the initial setting time, which can be seen in Table 4. On the other hand, the mortar with LWAs revealed a slight advancement of the velocity increase. The latter can be explained by the stiffness of the included aggregates, contributing to the formation of a stiff skeleton. However, the initial setting time remained close to the one of the reference mortars. Afterwards, a continued, logarithmic evolution of the velocity can be observed up to 18 h of curing for all mixtures, after which a plateau is reached. Before the attainment of the plateau, final setting takes place. A summary of the final setting times is given in Table 4. It can be seen that, opposing to the initial setting times, the final setting of mortars with SAPs occurs almost simultaneously with the reference material, which displays the effective preservation of the water inside the SAPs, not increasing the w/c ratio.

## 4. Conclusions

In this paper, the use of non-destructive elastic wave techniques to monitor cementitious materials with internal curing mechanisms was investigated. Three cement paste and mortar mixtures were investigated, being a reference blend without internal curing mechanism, a mixture with saturated LWAs and one with SAPs.

For the first time, AE was capable to monitor and distinguish the internal curing process, induced by SAPs or LWAs, demonstrated by the increased AE activity after final setting. By an analysis of the wave parameters, it was seen that the absolute energy and amplitude of the received waves varied during internal curing, indicating a variation in the process’ intensity. Additionally, AE allowed the understanding of the process of water release by the different additives. In case of saturated LWAs, the water was released at a high rate, while for SAP mixtures the activity continued up to several days of curing. The source of AE is related to water mobility through the porosity but mostly to SAP shrinkage and detachment from the internal surface of pore cavities. In addition, for the first time in literature, AE is shown capable to monitor the evaporation of water saturated specimens, something that should be further studied.

A comparison of AE monitoring between cement pastes and mortars was performed and displayed a different evolution of the wave parameters over time, for both SAP and LWA mixtures. In case of cement pastes, a gradual increase in absolute energy and amplitude was noticed from the onset of internal curing onwards, after which an opposite decreasing trend was seen. For mortar mixtures, these parameters increased rapidly, i.e., within two hours. A fairly constant maximum value was then maintained for SAP mixture, followed by a more gradual reduction in both parameters. These results illustrate the difference in water demand and development of capillary pressure between mixtures with and without aggregates.

Ultrasonic measurements disclosed the effect of SAPs and LWA on the microstructural development. Whereas the wave velocity indicated an earlier formation of solid connections within LWA mortars, caused by the inclusion of lightweight aggregates and thereby increasing the total aggregate content, a delay was observed for SAP inclusion. This delay is due to a diluted ion concentration by the absorption of ions by the SAPs during mixing, which slows down the hydration process and thus the initial setting time.

Lastly, the use of AE and ultrasound enables continuous monitoring of the various processes inside fresh cementitious materials. At the dormant stage, when the ultrasound velocity shows no variation, AE captures the settlement activity. When the silent period in AE comes and AE does not supply much information, ultrasound shows vividly the developments related to setting. Afterwards, when ultrasound velocity seems to converge between different media, AE starts again to supply detailed information and clearly distinguishes between reference, LWA and SAPs. Therefore, the combination of elastic waves techniques provides an affordable and relatively easy way to receive information on the microstructure that would be difficult to obtain in any other way.

## Figures and Tables

**Table 1:** Mixture compositions of reference, SAP and LWA mortar (kg/m^3^).

Mixture	Cement	Water	Superplasticizer	SAPs	LWAs	Sand
Reference	580	203	2.32	-	-	1160
SAP	580	233.16	2.32	1.16	-	1160
LWA	580	233.16	2.32	-	30.16	1160

**Table 2:** Density (g/cm³), flexural strength (MPa) and compressive strength (MPa) of mortar mixtures.

	Density (g/cm³)	Compressive Strength (MPa)
Reference	2.17 ± 0.01	77.3 ± 1.3
SAP	2.16 ± 0.04	72.4 ± 2.8
LWA	2.11 ± 0.01	61.6 ± 5.7

**Table 3:** Initial weight (g), saturated weight (g) and water uptake (g) of reference, SAP and LWA samples.

	Initial Weight (g)	Weight after Saturation (g)	Water Uptake (g)
Reference	517.33	530.90	13.57
SAP	512.55	525.02	12.47
LWA	478.24	497.02	18.78

**Table 4:** Initial and final setting time of reference, SAP and LWA mortars, based on two replicates per mixture.

	Initial Setting Time (h)	Final Setting Time (h)
Reference	3.38 ± 0.16	7.64 ± 0.38
SAP	4.02 ± 0.30	7.44 ± 0.10
LWA	3.60 ± 0.35	7.21 ± 0.13

## Data Availability

The data of this study is available upon request, where justified, by e-mail to the corresponding author.
